# Combining thermal, tri-stereo optical and bi-static InSAR satellite imagery for lava volume estimates: the 2021 Cumbre Vieja eruption, La Palma

**DOI:** 10.1038/s41598-023-29061-6

**Published:** 2023-02-04

**Authors:** Simon Plank, Alina V. Shevchenko, Pablo d’Angelo, Veronika Gstaiger, Pablo J. González, Simone Cesca, Sandro Martinis, Thomas R. Walter

**Affiliations:** 1 German Aerospace Center (DLR), Earth Observation Center, 82234 Oberpfaffenhofen, Germany; 2 GFZ German Research Centre for Geosciences, Telegrafenberg, 14473 Potsdam, Germany; 3 Instituto de Productos Naturales y Agrobiología (IPNA-CSIC), La Laguna, Spain

**Keywords:** Natural hazards, Solid Earth sciences

## Abstract

Determining outline, volume and effusion rate during an effusive volcanic eruption is crucial as it is a major controlling factor of the lava flow lengths, the prospective duration and hence the associated hazards. We present for the first time a multi-sensor thermal-and-topographic satellite data analysis for estimating lava effusion rates and volume. At the 2021 lava field of Cumbre Vieja, La Palma, we combine VIIRS + MODIS thermal data-based effusion rate estimates with DSMs analysis derived from optical tri-stereo Pléiades and TanDEM-X bi-static SAR-data. This multi-sensor-approach allows to overcome limitations of single-methodology-studies and to achieve both, high-frequent observation of the relative short-term effusion rate trends and precise total volume estimates. We find a final subaerial-lava volume of $212\times {10}^{6}\pm 13\times {10}^{6}\; \text{m}^{3}$ with a MOR of 28.8 ± 1.4 m^3^/s. We identify an initially sharp eruption-rate-peak, followed by a gradually decreasing trend, interrupted by two short-lived-peaks in mid/end November. High eruption rate accompanied by weak seismicity was observed during the early stages of the eruption, while during later stage the lava effusion trend coincides with seismicity. This article demonstrates the geophysical monitoring of eruption rate fluctuations, that allows to speculate about changes of an underlying pathway during the 2021 Cumbre Vieja eruption.

## Introduction

Quantification of the scale and dynamics of effusive eruptions is a key for hazard assessment and volcano activity extrapolations. In the long-term, global compilations of eruption volumes and eruption rates allow development of predictive studies on large-scale (e.g., Crips and coauthors^1^). Closer studies at individual volcanoes revealed that eruption rates may vary significantly through time, increase, decrease, or fluctuate^2^. The temporal behavior may be complex, which is why a major challenge in volcanic hazard assessment is to understand changes in the eruptive style, possibly associated with magma viscosity, gas loss and external properties such as conduit geometry^3^ and magma overpressure conditions^4^. As will be shown in this work, satellite remote sensing may be key to identify volume rate changes and hence better understand changing dynamics and hazards of an ongoing eruption.

In basaltic systems, such as the Cumbre Vieja, La Palma, information about the lava effusion rate during a volcanic eruption is very important as it is a major controlling factor of the lengths of a lava flow^5^. Therefore, the lava effusion rate is also a critical parameter for predictive models of lava flow aerial development^6^. Besides basaltic systems, also for more viscous, dome building lava compositions, information about the effusion rate are crucial for the understanding of the potential of a lava dome for explosive collapse^7^.

### Estimation of lava effusion rates by means of satellite remote sensing

Main benefits of satellite remote sensing techniques are especially more evident where the area of interest (AOI) cannot be safely and immediately accessed, so that it is difficult to gain in situ information, as well as during large and rapidly evolving eruptive events, as was the case during the 2021 Cumbre Vieja, La Palma volcanic eruption. Satellite-based volcano monitoring often relies on thermal, optical and Synthetic Aperture Radar (SAR) data analysis.

Thermal Earth Observation (EO) provides valuable information for estimating the lava effusion rate^8^ and has been a well-established technique for volcano monitoring since the early 1980s, beginning with the NASA Land Remote Sensing Satellite (Landsat) Thematic Mapper (TM) series and the Advanced Very High Resolution Radiometer (AVHRR) onboard the National Oceanic and Atmospheric Administration (NOAA) satellites^9,10^.

Important developments in automated thermal hotspot detection approaches are based on the Moderate Resolution Imaging Spectrometer (MODIS) provided by the Middle InfraRed Observation of Volcanic Activity (MIROVA) system^11^ and the MODIS volcano detection algorithm (MODVOLC)^12^. The high capabilities for thermal anomaly detection of the MODIS successor mission Visible Infrared Imaging Radiometer Suite (VIIRS) sensor were confirmed by^13,14^. Other automated hotspot detection systems such as HOTVOLC^15^ and HOTSAT^16^ enable volcanic activity analysis in near real-time as they are based on the high temporal resolution data from geostationary satellites (e.g., the Geostationary Operational Environmental Satellite (GOES) and Meteosat). Thermal satellite imagery has been used to investigate a variety of thermal volcanogenic emitting phenomena, such as lava lakes^17^, active lava flows^14^ and lava domes^18^.

Harris and Rowland as well as Harris and coauthors give a comprehensive review on the relationship between effusion rates and thermal emission of lava flows, and how to derive lava effusion rates from thermal satellite imagery^5,19^. As described in detail in Coppola and coauthors^20^, based on the original heat balance approach of Pieri and Baloga^21^, two main approaches for lava effusion rate estimation from thermal satellite data have been reported in literature: the thermal infrared (TIR) data method^22,23^, and the mid infrared (MIR) data technique^24^.

Here, the following terms are used as defined in Harris and coauthors^23^: The *effusion rate* is the instantaneous rate at which lava is erupted at any time. The *mean output rate* (MOR) is the entire erupted lava volume (after the end of the eruption) divided by the total eruption duration. The *time-averaged discharge rate* (TADR) is the lava volume emplaced averaged over a given time period. According to Wright and coauthors as well as Harris and coauthors, for satellite data-based analysis of effusion rates the TADR is considered as the most suited method as satellite sensors measure changes in lava volume not over the whole eruption duration but over a given time period prior to each satellite image acquisition^22,23^.

Examples for some recent eruptions studied with thermal EO data-based TADR estimates are the 2014–2015 Holuhraun eruption^20^, the 2018 eruption and sector collapse at Anak Krakatau^25^ or the 2018 Kīlauea volcano eruption^14^.

High (HR) and very high-resolution (VHR) optical satellite imagery are ideally suited for the detailed analysis of, e.g., lava flows and pyroclastic density currents^26^. Optical stereo data enables the generation of an up to date digital surface model (DSM) of the lava flow topography. The Advanced Spaceborne Thermal Emission and Reflection Radiometer (ASTER) can acquire stereoscopic images at 15 m spatial resolution for deriving DSMs. The time difference between the two along-track ASTER images is a few seconds only^27^. Modern sensors such as Pléiades with its tri-stereo acquisition applicability enable the generation of a detailed DSM of the volcano surface. For example, Bagnardi and coauthors compared topographic information derived from post-eruptive Pléiades imagery and pre-eruptive TanDEM-X data to measure the erupted lava area, volume, and the MOR of the 2014–2015 Fogo Volcano eruption^28^.

In contrast to optical and thermal sensors is SAR the only system that provides useful information of the Earth’s surface almost completely independent of weather and daylight and also during explosive eruption events when the visibility and applicability of optical and thermal sensors are limited by meteorological or volcanic ash clouds, respectively. Analysis of SAR amplitude data is a well-established tool for volcano monitoring also when major changes occur on the surface of the volcano, e.g. due to explosive eruptions^29,30^ and allowed the monitoring of the aligned craters at Cumbre Vieja^31^. SAR interferometry (InSAR) can be applied for measurement of slow terrain motion in unvegetated snow free areas^32^. However, classical, repeat-pass InSAR cannot be applied on sites of strong surface changes between the two SAR acquisition from which the interferometric phase is derived. Topographic differences calculated from two DSMs with one derived from a pre-eruption InSAR pair and a second one from a post-eruption InSAR pair gives information about the erupted lava volume and about the MOR^33,34^. However, for detailed TADR estimates, more information, i.e. also information about the lava flow topography during an ongoing eruption are required. Here, bi-static InSAR data acquisitions are very useful. For instance, in bi-static mode the mission TerraSAR add-on for Digital Elevation Measurements (TanDEM-X) provides two SAR images acquired over the same area at the same time (i.e. there is no temporal de-correlation of the interferometric phase), which enable the generation of an up to date DSM of the study site^35^. For example, Poland used a time series of TanDEM-X acquisitions and generated differential DSMs to measure the TADR of subaerial lava at Kīlauea Volcano, Hawai‘i^36^.

Hence, multi-sensor remote sensing, i.e., a combination of data from different Earth observation disciplines can make a significant contribution to the understanding of volcanic processes^37^. Example studies are described by Walter and coauthors investigating the 2018 flank collapse of Anak Krakatau^25^, Plank and coauthors analyzing the 2018 dome collapse at Kadovar^18^, and Shevchenko and coauthors describing the 2018–2019 eruption episode at Shiveluch volcano^38^.

In our study, we follow a multi-sensor data approach and describe for the first time a combination of thermal, tri-stereo optical and bi-static InSAR satellite imagery for analyzing lava effusion rates and volume. The methods are described in detail in “Methods” section. We investigated the 2021 Cumbre Vieja, La Palma eruption, which is briefly described in the next section.

### The 2021 Cumbre Vieja, La Palma eruption

**Figure 1:**
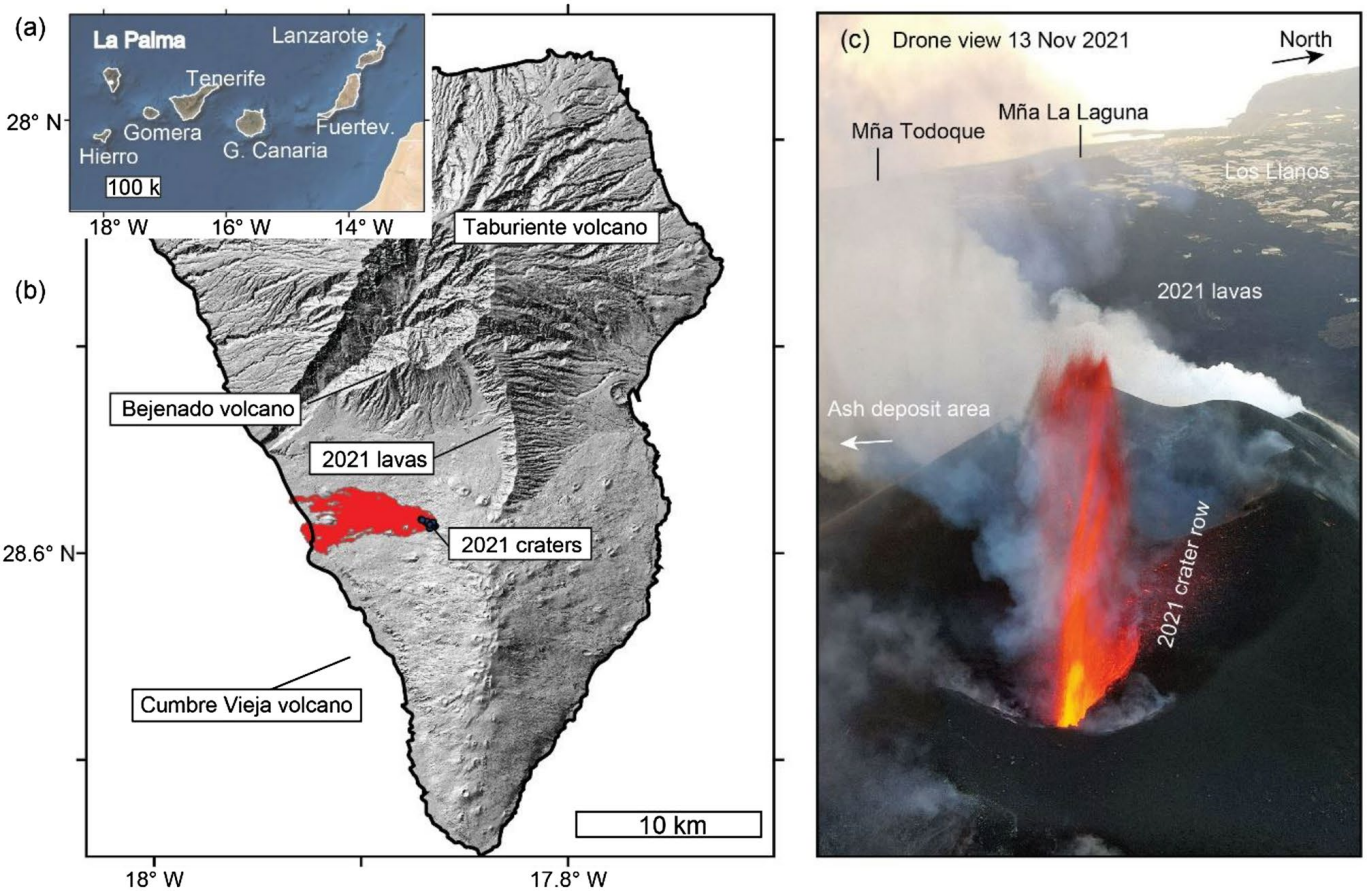
Overview map of the Canary Islands with La Palma in the NW (**a**). Topographic hillshade of La Palma with the 2021 Cumbre Vieja volcano lava field (**b**). Drone view from the active crater area over the lava field towards the western coast of La Palma (**c**). Drone image taken by us during field work on November 13, 2021. Map created using the GMT/MATLAB Toolbox (https://agupubs.onlinelibrary.wiley.com/doi/10.1002/2016GC006723).

On September 11, 2021, began a seismic swarm that gradually intensified over the next days^39^. This seismic swarm indicated a magma pathway that propagated along the Cumbre Vieja rift zone of La Palma. Finally, on September 19, 2021, the *Tajogaite* eruption began by the opening of an 800 m long fissure on the mid-western flank of Cumbre Vieja, more precisely in the area of Hoya de Tajogaite, El Paso. The eruption intensified over the next weeks and was characterized by lava fountaining from multiple vents aligned in direction NW–SE^31^, with sometimes four to five vents being active simultaneously, Strombolian explosions, and advancing lava flows towards the western coast of La Palma Island (Fig. 1). Dense geophysical and geochemical monitoring allowed recording temporal changes in high quality^39^ and assessment of possible volcano-tectonic control^40^. The lava flows entered into the ocean from September 28 onwards and initiated the formation of three lava deltas that eventually connected to one large and one smaller lava delta. Finally, the lava flows were regularly traced by the Copernicus Emergency Management Service (EMS), yielding a lava area of over 12.4 km^2^. Thereby, around 3000 houses were destroyed and over 7000 people were displaced^41–43^. The eruption had a duration of 12 weeks, which is four times of the duration of the last eruption in 1971. Locally lava flows exceeded 30 m thickness, but a systematic volume estimate and discharge estimation was not achieved. Due to the high impact hazards, the area was hardly approachable by field sensors, or localized drone flights allowing estimation of the dimension and evolution of surface changes. Therefore, we acquired and analyzed multiple sensor satellite data. During the initial dike intrusion period, multi-temporal differential SAR interferometry analysis of PAZ, TerraSAR-X/TanDEM-X, Cosmo-SkyMed and Sentinel-1 showed over 40 cm deformation along the line-of-sight of the ascending passes of the affected slope towards the sea^44^. To study the spatio-temporal evolution of lava flows and ash deposition, the following eruption was monitored by SAR amplitude and VHR multispectral imagery (e.g., Pléiades and GeoEye). For example, the Rapid Mapping service of Copernicus EMS was activated and produced 64 products (https://emergency.copernicus.eu/mapping/list-of-components/EMSR546). The spatio-temporal evolution of the lava flow area is well documented by the EMS maps. However, these maps do not answer the question about the temporal evolution of lava effusion rate and volume. In this study, we answered this question by investigating of multi-sensor satellite imagery.

## Results

We jointly analyzed thermal MODIS and VIIRS, VHR optical tri-stereo Pléiades and bi-static TanDEM-X SAR satellite data to investigate the 2021 Cumbre Vieja eruption event. Eventually we compare trend changes to a seismic catalogue acquired by independent methods, to develop a conceptual model explaining the observations. The data and methods are described in detailed in “Material and methods” section.

### Lava volume estimates from VHR satellite imagery

**Figure 2:**
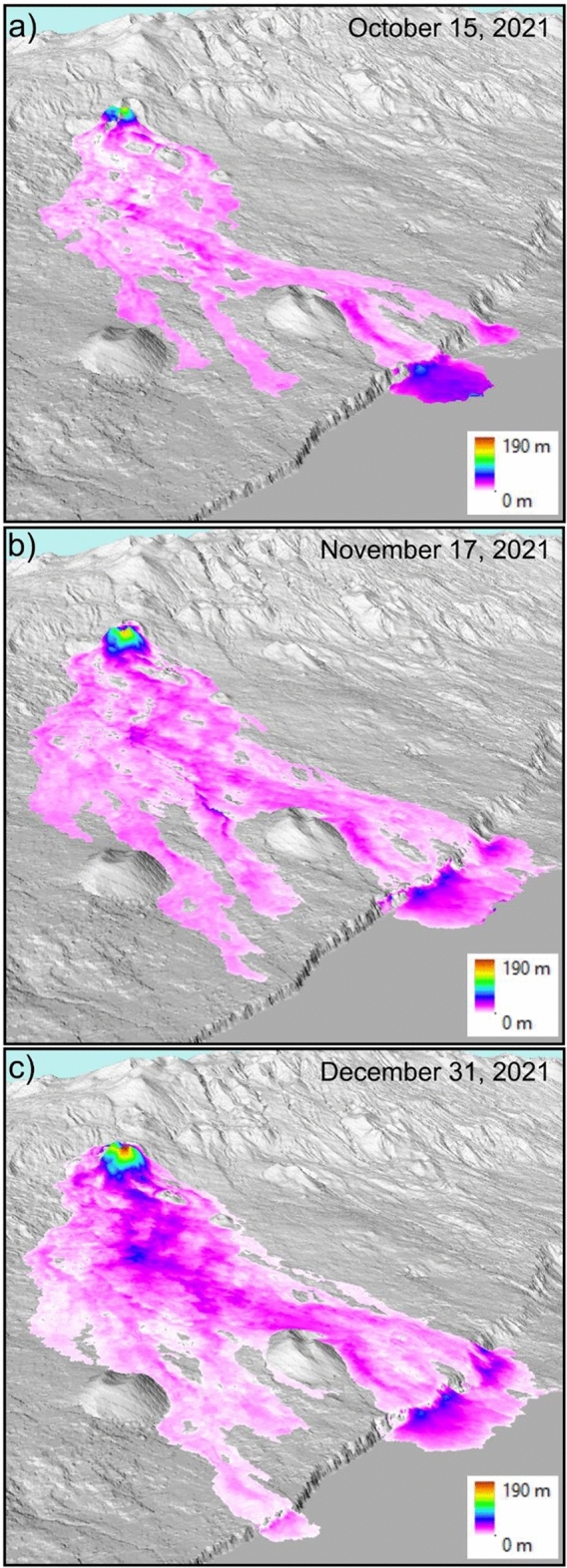
3D representation of the lava flow thickness maps derived from (**a**) TanDEM-X (October 15, 2021), (**b**) TanDEM-X (November 17, 2021) and (**c**) Pléiades (December 31, 2021) acquisitions in comparison with the pre-eruption LiDAR DSM (hillshade in background). North is to the left, image width is 3.6 km. Maps created using ESRI ArcScene 10.7.1.

Comparison of the TanDEM-X and Pléiades DSMs with the pre-eruption LiDAR DSM enabled us the generation of lava flow thickness maps (Fig. 2) and the calculation of the lava flow volumes (Table 1) for the dates of the VHR satellite data acquisitions. First, in order to correct for potential offsets, the TanDEM-X and Pléiades DSMs were compared with the pre-eruption LiDAR DSM at areas close to the lava flow, but unaffected by it. Second, the offset corrected TanDEM-X and Pléiades DSMs were cut to the lava flow areas according the corresponding Copernicus (EMS) mapping (cf. Table 2) and the lava volume were derived for these areas by difference calculation between the co-/post eruption TanDEM-X and Pléiades DSMs and the pre-eruption LiDAR DSM (cf. “DSM generation and lava volume estimates from optical tri-stereo data” and “DSM generation and lava volume estimates from bi-static TanDEM-X SAR data” sections for details). The uncertainty values reported are based on height differences of the satellite data-based DSMs to the pre-eruption LiDAR DSM within areas not affected by the eruption.

**Table 1:** Volume estimates based on VHR satellite data (*the software packages CATENA and Agisoft Metashape follow different processing schemes, cf. “DSM generation and lava volume estimates from optical tri-stereo data” section).

Dataset	Acquisition date	Measured lava volume
TanDEM-X bi-static	October 15, 2021	$101\times {10}^{6}\pm 13\times {10}^{6}\; \text{m}^{3}$
TanDEM-X bi-static	November 17, 2021	$158\times {10}^{6}\pm 18\times {10}^{6} \; \text {m}^{3}$
TanDEM-X bi-static	November 22, 2021	$171\times {10}^{6}\pm 20\times {10}^{6} \; \text {m}^{3}$
Pléiades tri-stereo (CATENA)*	December 31, 2021	$212\times {10}^{6}\pm 13\times {10}^{6}\; \text {m}^{3}$
Pléiades tri-stereo (Agisoft Metashape)*	December 31, 2021	$208\times {10}^{6}\pm 11\times {10}^{6} \; \text {m}^{3}$

**Table 2:** Silica content of previous eruptions at Cumbre Vieja^45,46^ and average values used in this study to investigate the 2021 eruption event.

Eruption event	Rock type and SiO_2_ in wt%	
	Tephrite	Basanit
1949 measurements	45.37 (min. 44.86, max. 45.77)	43.94 (min. 43.50, max. 44.51)
1971 measurements	44.49	43.27
Average values used in this study	44.93	43.61

**Figure 3:**
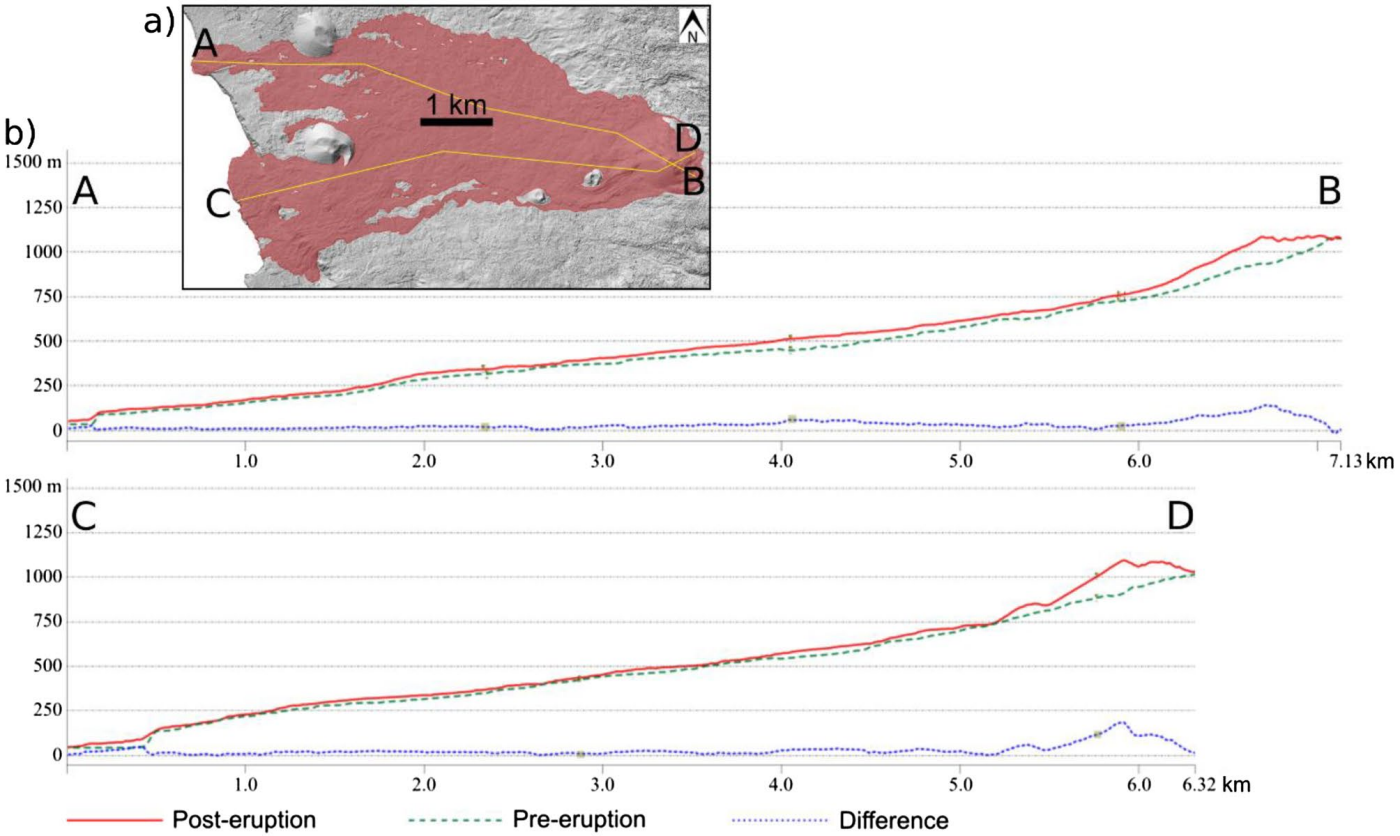
Hillshade visualization of the Pléiades DSM with the location of the profiles A-B and C-D on the final lava flow area (**a**). Profiles of the pre-eruptive and post-eruptive topography as well as the lava flow thickness derived by comparison of the Pléiades DSM (December 31, 2021) and the pre-eruptive LiDAR DSM (**b**). Map created using QGIS 3.28 https://www.qgis.org/.

Figure 2 also shows the formation of two lava deltas at the southern part of the lava flow (October, Fig. 2a), which then connected to one large delta (November, Fig. 2b). A third lava delta formed later at the northern part of the lava flow (December, Fig. 2c). Two profiles of the pre-eruption and post-eruption topography as well as of the lava flow thickness are shown in Fig. 3. The uphill vents area and the coastal lava deltas show the highest lava flow thickness.

### Lava effusion rate and volume estimates from thermal satellite imagery

**Figure 4:**
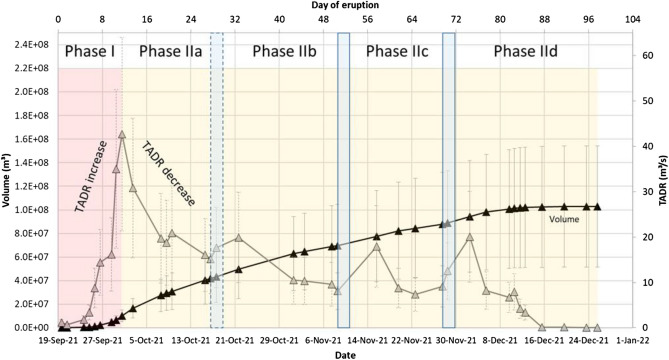
TADR (grey) and lava volume (black) estimates based on joint analysis of MODIS and VIIRS thermal EO data. The rising phase (I) of the effusion rate is marked in red color, the long waning phase (II) is marked in yellow color. The blue boxes mark the beginning of the phases IIb, IIc and IId. Compared to the later pulses, the one in Phase IIb is less strong and therefore marked with a dashed outline.

Figure 4 shows the TADR derived via the combined analysis of MODIS and VIIRS thermal imagery. This analysis is based on the empirical relationship between the radiative power measured by the thermal sensor over the lava field (volcanic radiative power, VRP) and the silica content of the lava, which regulates the viscosity and thereby the flowing properties of the lava^24^ (cf. “Lava effusion rate and volumes estimates from thermal satellite imagery” section for more details). The first 4 days of the eruption showed relatively low effusion rates of ~ 1.2 m^3^/s. But, from September 24, 2021 onwards and especially from September 27 onwards, a strong increase of the effusion rates up to values of 42.7 ± 21.3 m^3^/s were observed. Seismic data showed a reactivation of the 12–15 km cluster on September 27, 2021 (00:00 UTC), indicating the activation of a deeper magma source. Therefore, from that date onwards, we assume a change of the lava composition from tephrite to basanite as was observed in previous eruptions at La Palma (e.g. in 1949 and 1971^45,46^). This lava composition change comes along with a decrease of the lava viscosity (cf. Table 2). Change of lava composition, which causes a decrease of the viscosity, leads to an increase of the effusion rate^14,24^. For the TADR we used the silica content of tephrite in the beginning of the eruption and then replaced it by the silica content of basanite from September 27, 2021 onwards.

From beginning of October until beginning of December 2021 we observed an average effusion rate of 13.3 m^3^/s with peaks of increased effusion rates on October 21, November 15 and December 2. Then, the effusion rate continuously declined down to 0.1 m^3^/s from December 15 until the end of the observation period on December 25, 2021. The official eruption finish date was December 13, 2021. Thus, these very low eruption rates from December 15 until December 25 are either due to residual heat of the recent lava flow emplacement or that minor but non-zero material continued flowing in well insulated lava tubes.

Thus, we can identify two main phases, a rising phase during the first 2 weeks of the eruption (Phase I cf. Fig. 4), followed by a long waning phase (Phase II cf. Fig. 4) lasting ten to 12 weeks (depending on the end of the eruption, as mentioned above). The waning phase is interrupted by short pulses: A smaller increase of the effusion rate during mid-October (Phase IIb, dashed-outline blue box in Fig. 4), and two stronger pulses during mid-November (Phase IIc) and again in late November/beginning of December (Phase IId; blue boxes in Fig. 4).

Figure 4 also shows the cumulative lava volume (cf. “Lava effusion rate and volumes estimates from thermal satellite imagery” section) and its temporal evolution as estimated from thermal satellite imagery by computing the integral of the consecutive TADR estimates. We see a strong increase of the lava volume at end of September 2021 as shown in the TADR estimates. This is followed by an almost linear growing trend until December 15, when the lava volume reached a steady state (flattening of the volume curve). The thermal data estimates give a final erupted lava volume of $103\times {10}^{6}\pm 51\times {10}^{6}\; \text{m}^{3}$, which results in a MOR of 12.3 ± 6.1 m^3^/s for the period of high thermal activity (97 days) or a MOR of 13.9 ± 7.0 m^3^/s for the official eruption period (85 days and 8 h), respectively.

Considering the first 5 weeks of the waning phase (Phases IIa and IIb, cf. Fig. 4), we can apply a simple mathematical model ($TADR=-17.91 \frac{{\text{m}}^{3}}{\text{s}}\times \mathrm{ln}\left(d\right)+77.422\frac{{\text{m}}^{3}}{\text{s}}$, with *d* representing the day of eruption) explaining the trend to 82.54%. Moreover, if we only consider these first 5 weeks of the waning trend (Phases IIa and IIb), we could predict the duration of the eruption with 88.23% confidence. I.e., based on only the information available during Phases IIa and IIb, we would expect the eruption to end after 75 days, which is 10 days earlier than the official eruption end (cf. previous paragraph). The reason for the underestimation of the eruption duration are the two short effusion rate pulses (in the Phases IIc and IId) during mid-November and again in late November/beginning of December. “Comparison with seismic observations” section discusses the Phases IIc and IId in detail and compares the TADR measurements with seismic observations.

### Lava volume estimates from multi-sensor satellite data

**Figure 5:**
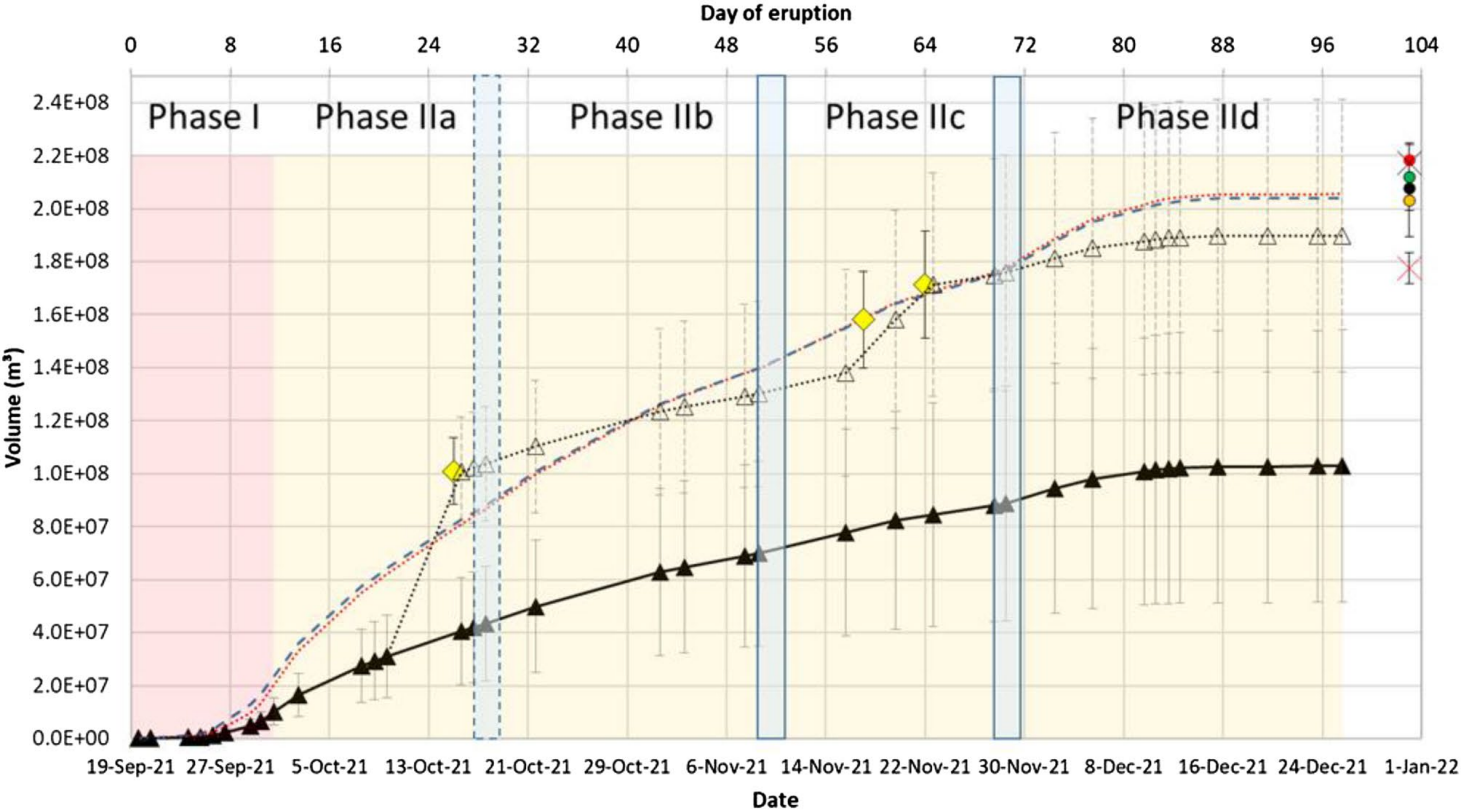
Lava volume evolution estimated from thermal data only (black filled triangles, same as Fig. 4), overlaid by calibrated thermal data-derived volume time series (open black triangles, black dotted line). TanDEM-X volume estimates (yellow diamonds) were used for calibrating the thermal volume time series. Our own final lava volume estimates by Pléiades tri-stereo DSM (green circle—CATENA, black circle—Agisoft Metashape). The blue dashed line simulates the original thermal data estimates but multiplied by a factor of 2. The red dotted line shows the thermal volume estimates when modelling an increased the silica content (cf. “Comparison of the different approaches” section). Copernicus EMS volume estimate based on Pléiades tri-stereo (February 3, 2022, red circle). Pléiades data-based volume estimates by^42^ (orange circle). Post-eruptive drone data-based volume measurements by^47^ are shown by the red cross (only lava) and black cross (lava and proximal fallout) (cf. “Comparison with independent measurements” section).

Based on the VHR TanDEM-X lava volume measurements, we calibrated the thermal estimates of Fig. 4. This calibration was performed by replacing the thermal volume estimates by the next available VHR TanDEM-X lava volume measurement acquired during the eruption (cf. “Methods” section “Lava volume estimates from multi-sensor satellite data”). This calibrated time series combines information about short-term eruption rate changes, derived from the high frequent thermal observations, with precise estimates of the absolute volume (TanDEM-X) whenever available. Figure 5 shows the original and the calibrated thermal data-based volume time series together with the volume measurements derived from the VHR TanDEM-X and Pléiades satellite data. The calibrated thermal estimates slightly still underestimate the final lava volume measured by Pléiades. But, considering the relatively high uncertainty of the empirical thermal methodology (cf. “Lava effusion rate and volumes estimates from thermal satellite imagery” section), we see that the calibrated thermal estimates are within the uncertainties of the Pléiades estimates.

## Discussion

### Comparison with independent measurements

Copernicus EMS analyzed two post-eruptive Pléiades tri-stereo datasets (acquired on December 31, 2021, which we also used in our study, and additionally on February 3, 2022), resulting in a final lava volume estimate of $216\times {10}^{6} \; \text {m}^{3}$ or $218\times {10}^{6} \; \text {m}^{3}$, respectively (Fig. 5)^42^. The EMS measurements are very close to our measurements of the final lava volume $212\times {10}^{6}\pm 13\times {10}^{6} \; \text{m}^{3}$ (extracted using the CATENA DSM) and $208\times {10}^{6}\pm 11\times {10}^{6}\; \text{m}^{3}$ (extracted using the Agisoft Metashape DSM) and confirm the reliability of our results regarding the final erupted lava volume. Belart and Pinel estimated a little bit lower total volume $203.3\times {10}^{6}\pm 13.9\times {10}^{6}\; \text {m}^{3}$ using the same Pléiades tri-stereo datasets acquired end of December 2021^48^. Naturally, all satellite data-based measurements of the erupted lava volume can only capture the amount of lava that was deposited on land, i.e. the submarine part of the lava volume can be captured only via bathymetry measurements.

Copernicus EMS only reports about the final erupted (subaerial) lava volume. In contrast to this, in our study we analyzed in addition to the post-eruptive Pléiades tri-stereo dataset, also a series of co-eruptive TanDEM-X bi-static datasets and a dense time series of thermal EO data (MODIS and VIIRS), which enabled us to measure not only the final erupted lava volume, but also its spatio-temporal evolution.

Using a structure-from-motion approach, Civico and coauthors generated a detailed (0.2 m resolution) post-eruptive DSM of the 2021 Cumbre Vieja lava flows based on drone imagery^47^. The authors report for the subaerial deposit of lava flows and proximal fallout a volume of $217\times {10}^{6}\pm 6.6\times {10}^{6}\; \text {m}^{3}$ and for the subaerial lava flows alone a volume of $177\times {10}^{6}\pm 5.8\times {10}^{6}\; \text {m}^{3}$ (cf. Fig. 5). The first mentioned lava volume matches well with our Pléiades data-based measurements of the final lava volume of $212\times {10}^{6}\pm 13\times {10}^{6}\; \text {m}^{3}$ (CATENA DSM) and $208\times {10}^{6}\pm 11\times {10}^{6}\; \text {m}^{3}$ (Agisoft Metashape DSM) and is also very close to the aforementioned measurements of Copernicus EMS. The second mentioned volume of Civico and coauthors^47^, i.e. the volume of the lava flows alone, is ca. 15 to 18% lower than our or the Copernicus EMS estimates. All the methodologies for differential DSM based volume measurements, VHR optical satellite, bi-static InSAR or the drone measurements of Civico and coauthors^47^ rely on a good match of the pre-eruption and the co-/post-eruption DSM. For this precise information about areas not covered by ash deposits is required. This is much better possible with the very precise drone DSM compared to the lower resolution satellite data-based DSMs.

### Comparison of the different approaches

Each of the single EO methodologies (thermal, VHR optical tri-stereo, bi-static InSAR) applied in this study for lava volume estimates has their advantages and disadvantages. The advantage of the thermal EO is its very high observation frequency with in case of combining MODIS and VIIRS observations up to eight observations per day (for the location of La Palma), which enables the measurement and analysis of (relative) short-term eruption rate changes. The drawback with thermal EO data-based lava volume and TADR estimates is that cooling effects and crust growth at the lava surface lead to an underestimation of the total lava flow volume, especially when lava flows in tubes beneath a crusted surface which works as an isolator above the hot liquid lava^49^. With thermal EO high temperatures can only be measured at the vents and at fresh cracks. Consequently, thermal EO alone leads to an underestimation of the erupted lava volume.

VHR satellite data from optical or SAR sensors enables a more precise lava volume estimation, independently of the isolating effects of the lava crust. VHR optical stereo or tri-stereo EO allows in general very good DSM generation and therefore precise lava volume estimates. The aforementioned term “in general” is used as the dark surface of lava flows does not always provide a high enough contrast to find a dense network of matching points between the two or three image pairs (for stereo or tri-stereo acquisitions, respectively). Bagnardi and coauthors reported similar for Fogo Volcano, where a low density of matching points was found due to the low texture caused by volcanic ash cover^28^. The pre-processing image correction in Agisoft Metashape made it possible to solve this problem in the second approach. Another limitation of optical data for volcano monitoring is the requirement of clear sky conditions. The location of La Palma as an oceanic island and the huge amount of volcanic ash emitted during several phases of the eruption did not allow to realize a clear-sky tri-stereo acquisition during the eruption event, but only after the end of the eruption.

Bi-static InSAR EO provides good lava volumes estimates, with higher uncertainty as the VHR optical (tri-)stereo data, but unbiased in contrast to the thermal estimates. As SAR sensors provide information of the Earth’s surface independent of the weather or visibility conditions, we were able to acquire several useful TanDEM-X acquisitions during the eruption event. Besides some technical problems in December 2021, which did not allow the acquisition of more TanDEM-X bi-static datasets another limitation is that the current mission state of TanDEM-X does not allow a regular and systematic bi-static monitoring of all active volcanoes around the globe. All acquisitions have to be manually tasked and conflicts between different customers/data requestors have to be solved. This reduces the number of possible bi-static acquisitions over the AOI. Future missions such as Tandem-L would be a great step forward regarding an operational and global volcano monitoring.

The advantage of the multi-sensor approach for lava volume estimation presented in this study is that we combine the advantages of the single methods and overcome their limitations. The combined analysis of thermal, bi-static InSAR and (tri-)stereo optical data enables to get both, high frequent observation (to study the relative short-term effusion rate trends) and more precise estimates of the absolute lava volume.

Comparison of the calibrated to the original thermal volume estimates shows that the calibrated values are above the uncertainty (± 50%) of the original estimates (Fig. 5). This ± 50% of the radiant density was introduced by Coppola and coauthors^24^ in order to account for the effects that bulk rheology has on spreading rate of active lava (cf. “Lava effusion rate and volumes estimates from thermal satellite imagery” section). For the 2021 La Palma eruption analyzed in this study, a factor of 2 (i.e. + 100%) of the original thermal estimates, instead of the factor 1.5 (+ 50% uncertainty) as proposed by Coppola and coauthors^24^, would show a relatively good fit with the VHR (TanDEM-X and Pléiades) measurements (see blue dashed line in Fig. 5). With this factor 2 one would correct the aforementioned underestimation of the lava volume by thermal EO due to cooling and crust growth at the lava surface and the effects of lava flowing in tubes beneath a crusted surface. However, it is important to mention that this factor 2 is so far only valid for the 2021 La Palma eruption. More investigations of other eruption events (with combined thermal and VHR optical/InSAR data analysis) are necessary in future, before one should apply this factor 2 in general.

Assuming the calibrated thermal time series or better the multiplied by factor 2 thermal time series as a realistic volume estimate, we performed an inverse calculation of the Eqs. (1) and (2) to model the silica content ${X}_{{\text{SiO}}_{2}}$ (cf. red dotted line in Fig. 5) that would be necessary to get the same volume estimates as for the “factor 2” estimate (cf. the good match of the red dotted and blue dashed line in Fig. 5). The resulting silica content values of the inverse modelling are 50.0 wt% (before September 27, 2021 cf. “Lava effusion rate and volumes estimates from thermal satellite imagery” section) and 46.5 wt% (from September 27 onwards). The first aforementioned silica content value of the inverse modelling (50.0 wt%) is definitely too high compared to the laboratory silica content measurements of real lava samples of La Palma (cf. Table 2). The second silica content value of the inverse modelling (46.5 wt%) is also higher than the ones reported in Table 2, but it is at the maximum of the range measured by Castro and Feissel^50^. However, 46.5 wt% is definitely higher than the basanite-tephrite composition characterized by low SiO_2_ contents (< 45 wt%) that is typical amongst Cumbre Vieja’s most recent eruptions^45^. Consequently, it is not a matter of “wrong” or too low silica content values when estimating the lava volume evolution based on thermal EO data, but it is the fact, that the isolating effect of the growing crust at the lava surface and the phenomena of hot liquid lava flowing in tubes result in an underestimation of the total lava flow volume. Therefore, a multi-sensor approach as described in this study is necessary to get precise estimates of the lava volume together with detailed information about its temporal evolution.

### Comparison with seismic observations

Comparison of surface observations with seismic data have helped to better understand activity changes and interactions of adjacent systems^51,52^. Seismicity changes observed during the *Tajogaite* eruption on La Palma mainly occur at depths of 10–12 km or at 34 km, and are reflecting pressure changes at depth, which eventually may be responsible for the observed changes in eruption dynamics, eruption location and the propagation at the summit craters^31^. Similar relationships between eruption changes and crustal seismicity were proposed for Kīlauea^53^, for the 2014 Bárðarbunga eruption^54^, and for volcanoes in Kamchatka^51,52^. Possibly a pressure surge causes the communication between the deep and shallow processes, which during the *Tajogaite* eruption guided the differentiation of phases of activity^31^, where profound changes in activity occur during (i) multiple collapse of a crater wall and venting activity, and (ii) during development of new and clustered craters. According to the geomorphology and seismology comparison in Muñoz and coauthors^31^, the activity first shows initial linear craters above the erupting dike in NW–SE orientation, extension of the crater row, and finally a disaggregation of the craters due to a new dike intrusion. The transition of these phases is marked or preceded by pronounced seismic activity increases, and compares well to some of the phases we identify in this work.

**Figure 6:**
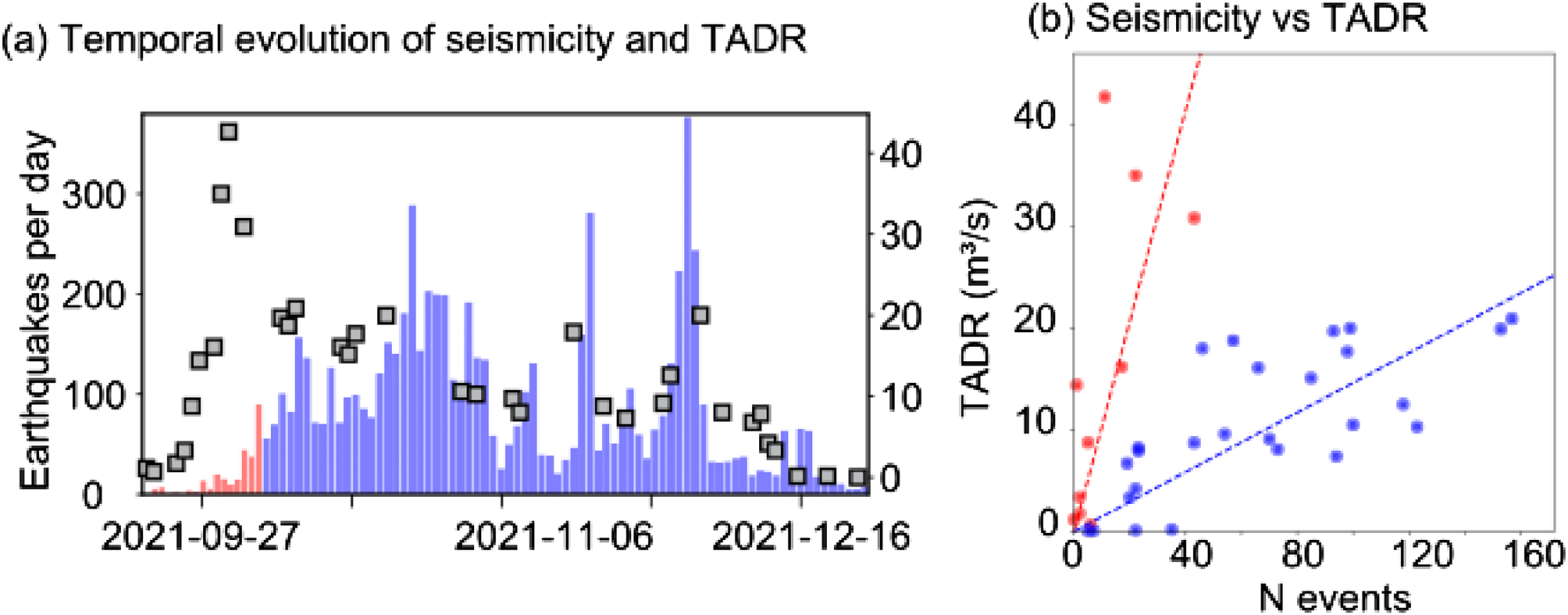
(**a**) Temporal evolution of seismicity and TADR. (**b**) Seismicity versus TADR. Two different trends can be observed: a diverging trend with an increase of effusion rate together with rather low seismicity (shown in red color) and a correlated trend after October 5, 2021 (shown in blue color). Pearson correlation-coefficients for the two time periods are 0.65 (red) and 0.73 (blue), respectively.

Figure 6 compares the temporal evolution of the lava effusion rate (TADR) with seismic measurements (earthquake counts). Only shallow earthquakes with a depth of ≤ 15 km were considered, thus any seismicity at a deeper level (such as the 34 km seismic zone) is excluded in this analysis. The ≤ 15 km depth range is considering the depth of the shallow magma reservoir as well as any pathways to the surface. Earthquake events at the shallow cluster, occurring within 1 day at the timing of the TADR estimate (12 h before to 12 h after) were considered in the analysis. We identify an initially strong diverging trend, where the lava effusion rate strongly increases whereas the seismicity is rather low during onset of the eruption. After October 5, 2021 (00:00), this anticorrelated behavior inverts to a correlated behavior in the long-term, where lava effusion rate declines are associated with weakened seismicity. However, particular of interest are two periods in the late evolution stage of the eruption, in mid-November and in end-November/beginning of December. Contrary to the aforementioned trend, these two periods show again much higher effusion rates also compared to the periods before, between them and after. Also Figs. 4 and 5 shows for these periods a deviation from the expected lava flow volume trend (Phases IIc and IId).

This increase of the effusion rate is also reflected by an increase of seismic activity within these periods. This phenomenon is visible relatively well in the mid-November period and very imminent in the late-November/early-December period.

Interpreting those effects and couplings are important. The lack of correlation during early stages of the eruption may indicate that the large TADR emission occurs along a magma pathway that is already open. In fact, independent studies suggest that the bulk of deformation occurred prior to the eruption onset^39^. Following correlated gradual decline and correlated short peaks in seismicity and TADR indicate a coupling of eruption rates to the opening of the magma pathway. It is worth noting, that the observed changes in effusion rate and thermal radiant energy in mid-November and late-November/early-December could be correlated with the appearance of a new fracture system and isolated eruptive vents deviating from the main summit cone^40,55^. This underlines the value of integrated and multi-sensor monitoring to understand the dynamics of basaltic eruptions.

## Conclusions

Information about the lava effusion rate during an effusive eruption is very important as it is a major factor controlling the lengths of a lava flow. This study described for the first time a multi-sensor satellite data approach for estimation of lava effusion rates and volume as well as their temporal evolution. We jointly analyzed time series information from thermal MODIS and VIIRS, VHR optical tri-stereo Pléiades and bi-static TanDEM-X SAR data. By combining their advantages, our multi-sensor approach overcomes the limitations of the single EO methods, which are for example underestimation of the absolute lava volume by thermal EO due to lava crust formation and lava flowing in tubes, the need for clear-sky conditions for thermal and optical EO, the relatively long repeat cycle of VHR satellite sensors. We used the precise VHR lava volume measurements to calibrate the more frequent thermal data-based volume estimates. Consequently, the multi-sensor data analysis enables to get both, high frequent observation (to study the relative short-term effusion rate trends) and precise estimates of the absolute lava volume. We investigated the 2021 *Tajogaite* eruption at Cumbre Vieja, the largest recorded eruption event at La Palma Island. The final subaerial lava volume was estimated to $212\times {10}^{6}\pm 13\times {10}^{6}\; \text {m}^{3}$, which gives a mean output rate (MOR) of 28.8 ± 1.4 m^3^/s. Independent measurements by Belart and Pinel^48^, Civico and coauthors^47^ and by Copernicus EMS^42^ confirm our estimates of the total erupted lava volume. We identify phases of eruption by short term pulses of higher effusion rates. The initial phase is accompanied by weak seismicity only. Periods of strong lava effusion rates in the late eruption phase, however, coincide with strong seismicity and are contrary to the general declining trend and deviate from the expected lava volume trend. These may reflect changes in the underground magmatic plumbing system observable via satellite data analysis. Results show the added value of volumetric lava flow monitoring and underlines that eruption rate fluctuations may be geophysically monitored, allowing to speculate about changes of an underlying pathway during the 2021 Cumbre Vieja eruption.

## Material and methods

### Data

VHR optical tri-stereo Pléiades, bi-static TanDEM-X SAR and thermal MODIS and VIIRS satellite data were jointly analyzed to investigate the 2021 Cumbre Vieja eruption event.

#### Optical tri-stereo imagery

The location of La Palma as an oceanic island, regular cloud or ash coverage did not allow an acquisition of a completely clear sky Pléiades tri-stereo dataset during the eruption. The first clear sky Pléiades tri-stereo dataset (acquired on December 31, 2021, cf. Table 3) could be realized after the end of the eruption. This acquisition was the only one which captured the entire lava field without any disturbances by meteorological or volcanic ash clouds.

**Table 3:** Datasets used.

Dataset	Acquisition date (time)	Corresponding Copernicus EMS lava map^a^
MODIS	September 19–December 25, 2021	–
VIIRS	September 19–December 25, 2021	–
TanDEM-X bi-static	October 15, 2021 (19:14 UTC, ascending orbit)	Numb. 22, October 15, 2021
TanDEM-X bi-static	November 17, 2021 (19:14 UTC, ascending orbit)	Numb. 53, November 20, 2021
TanDEM-X bi-static	November 22, 2021 (07:16 UTC, descending orbit)	Numb. 54, November 22, 2021
Pléiades tri-stereo	December 31, 2021 (12:09 UTC)	Numb. 63, December 17, 2021
LiDAR DSM	2009	–

^a^https://emergency.copernicus.eu/mapping/list-of-components/EMSR546

#### TanDEM-X bi-static SAR data

Three TanDEM-X bi-static acquisitions could be realized over the Cumbre Vieja lava field during the eruption event: October 15, November 17 and November 22 (cf. Table 3). Unfortunately, temporally technical problems at the satellite TanDEM-X in December 2021 did not allow further acquisitions during the eruption event. The TanDEM-X satellite constellation consists of the two SAR satellites TerraSAR-X and TanDEM-X flying in a tandem constellation with 120 to 500 m baseline. In case of a bi-static acquisition, one of these satellites transmits a radar signal to the Earth’s surface and both satellites receive the SAR backscatter simultaneously at slightly different incidence angles. This enables the generation of SAR interferograms with high coherence of all land surfaces as no changes occur on ground between the two simultaneously acquired SAR images^35^.

#### Pre-eruption LiDAR DSM

A 5 m spatial resolution DSM of La Palma Island created by the Centro Nacional de Informacion Geografica from airborne LiDAR measurements carried out in 2009 was available. This LiDAR DSM serves as pre-eruption topographic information source. The LiDAR DSM and all VHR satellite data were in or transformed to WGS84 (UTM zone 28N).

#### Thermal satellite imagery

Daily MODIS (flying on the Aqua and Terra satellites) and VIIRS (flying on NOAA-20 and Suomi-NPP) data over the Cumbre Vieja lava field from September 19, 2021 (beginning of the lava flow eruption) until December 25, 2021 (last thermal hotspot detected at lava flow area) were analyzed. All 4 satellites have a joint revisit time of at least eight overflights per day over the study area. The thermal hotspots were detected using the 375 m resolution VIIRS bands I4 (MIR, center wavelength λ = 3.74 µm) and I5 (TIR, λ = 11.45 µm) and the 1 km resolution MODIS MIR bands 21/22 (λ = 3.96 µm) and TIR band 31 (λ = 11.03 µm). The hotspot data were derived from the Fire Information for Resource Management System^56,57^.

Information about the chemical lava compositions from previous eruptions at Cumbre Vieja, La Palma in 1949 and 1971 were derived from Hernandez-Pacheco and Valls^45^ and Klügel and coauthors^46^. It is assumed that the lava composition of the 2021 eruption event is very similar to that of previous eruptions: the eruption started with more viscous tephrite and then later by activating a deeper magma source changing to less viscous basanite. We calculated the average of the silica content (SiO_2_) of the aforementioned previous eruptions (Table 2) and used these values for the thermal satellite data-based lava volumes estimates (cf. “Lava effusion rate and volumes estimates from thermal satellite imagery” section).

### Methods

#### DSM generation and lava volume estimates from optical tri-stereo data

The Pléiades tri-stereo dataset consists of three scenes acquired at different looking angles during one overflight over the Cumbre Vieja lava field. The Pléiades tri-stereo data were processed using (1) the Semi-Global-Matching algorithm implemented in the DLR software environment CATENA^58^ and (2) Agisoft Metashape v1.8. to derive a 0.50 m resolution DSM of the lava field and its surroundings as of December 31, 2021 testing the two different approaches.

Dark surfaces such as lava fields do not always provide high enough contrast. The same applies for ash covered areas due to the similar and smooth texture of the ash^28^. This partly led to missing matching points between the single tri-stereo acquisitions, causing small gaps in the DSM derived with CATENA. We filled these few small gaps within the Pléiades DSM by inverse distance weighting (IDW) interpolation of the neighborhood height information values. However, the DSM generated in Agisoft Metashape has not gaps because we used the pre-processing contrast correction provided by the software.

Prior to lava volume estimation via differencing of post-eruption the CATENA Pléiades DSM and the pre-eruption LiDAR DSM (cf. “Pre-eruption LiDAR DSM” section), an area in the southern neighborhood of the lava field, where almost no volcanic ash was deposited, was selected. Differencing of the post- and pre-eruption DSMs showed an average vertical offset of − 5.5 m for that area, where actually no topographic changes occurred during the eruption event. Consequently, this offset was added to the post-eruption DSM in order to guarantee the same base height within unaffected areas. Cross-check within the unaffected areas gives an RMSE of the offset corrected DSM of 1.1 m. The offset of the Agisoft Metashape Pleiades DSM was measured in eight different areas around the lava flow with the final RMSE of 0.9 m. This value was later distributed over the area of the lava field to further estimate the lava volume error.

Next, both Pléiades DSMs (CATENA and Agisoft Metashape) as well as the pre-eruption LiDAR DSM were cut to the extent of the final lava flow area as mapped by the corresponding Copernicus (EMS) map (cf. Table 3). Then, the lava flow cut pre-eruption LiDAR DSM was subtracted from each lava flow cut post-eruption the Pléiades DSMs to compute the final lava flow volume.

#### DSM generation and lava volume estimates from bi-static TanDEM-X SAR data

The three bi-static TanDEM-X datasets were processed with the ENVI^®^ SARscape^®^ software to derive from the Coregistered Single look Slant range Complex (CoSSC) TanDEM-X data DSMs via SAR interferometric processing. The processing workflow includes interferogram generation and flattening, phase filtering (using the Goldstein phase filter) and unwrapping (using the Minimum Cost Flow Approach), phase to height conversion and geocoding. The pre-eruption LiDAR DSM was used as base information for the bi-static TanDEM-X DSM generation. All TanDEM-X DSMs were processed to 5 m spatial resolution. The heights of ambiguity are 72.05 m (October 15), 89.08 m (November 17) and − 24.79 m (November 22).

Geolocation mismatches of the TanDEM-X DSMs compared to the pre-eruption LiDAR DSM were corrected by georeferencing via manual ground control points. Vertical offset measurements to the pre-eruption LiDAR DSM and the lava volume calculation were done for all three TanDEM-X DSMs as described in “DSM generation and lava volume estimates from optical tri-stereo data” section) for the CATENA Pléiades DSM. The RMSE are 1.8 m, 1.7 m and 1.9 m for the TanDEM-X acquisitions October 15, November 17 and November 22, respectively. The georeferenced and offset corrected TanDEM-X DSMs and the pre-eruption LiDAR DSM were cut to the lava flow extents as mapped by Copernicus EMS at the corresponding dates (cf. Table 3). Finally, the lava volumes were derived via differencing of the cut corrected TanDEM-X DSMs from the cut pre-eruption LiDAR DSM.

#### Lava effusion rate and volumes estimates from thermal satellite imagery

The VIIRS and MODIS data were analyzed as follows: (1) only data with low scan angles were considered, as high satellite zenith angle strongly influence the reliability of volcanic hotspot detection, inducing possibly distortion effects^11^. MODIS data with zenith scan angle values < 40.00° were selected^11^. VIIRS data acquired at a scan angle ≤ 31.59° were considered as this scan angle region corresponds to the first aggregation region of VIIRS, where three native pixels are aggregated along the scan direction to form one data sample in the Level 1 image^56^. (2) In order to allow analysis of potential cloud cover, only day time MODIS and VIIRS data were considered. The influence of reflected sunlight is very low in MIR region, particularly at high temperatures related to great effusive events like the 2021 La Palma eruption, and may not be considered^8^.

Then, only images showing clear sky condition over the lava field were selected to avoid underestimation of the lava effusion rate due to (partly) cloud or volcanic aerial ash over the study site.

Next, the volcanic radiative power (VRP) was calculated for the MODIS and VIIRS hotspots using the MIR approach described by Wooster and coauthors^59^. This MIR approach assumes that the measured heat flux is just related to lava portions having a radiating temperature above 600 K. This approach is especially valid for hot bodies with an integrated temperature between 600 and 1500 K. According to Wright and coauthors, these conditions apply to most active lava bodies, such as lava flows^60^.

Then, the total VRP over all hotspots detected within each single daytime (see above) overflight of MODIS and VIIRS were calculated. Next, the overflight with the maximum total VRP per day was selected and considered in the following processing steps. We selected the maximum total VRP per day in order to compensate for potential missing detection of hotspots due to thin ash or meteorological clouds that were missed in the aforementioned visibility check of the satellite imagery.

According to the empirical approach of Coppola and coauthors, the TADR and the erupted lava volume were estimated^24^. This approach directly links the TADR with the VRP (Eqs. (1), (2)):

1 $$TADR= \frac{VRP}{{c}_{rad}}$$

with c_rad_ (in J/m^3^) is called radiant density

2 $${c}_{rad}=\frac{6.45\times {10}^{25}}{{\left({X}_{{\text{SiO}}_{2}}\right)}^{10.4}}$$

c_rad_ represents the empirical relationship between radiant and volumetric flux for the analyzed thermal emitting lava. ${X}_{{\text{SiO}}_{2}}$ is the silica content (normalized to 100%) of the erupted lava investigated (cf. Table 2).

According to Coppola and coauthors, an uncertainty of ± 50% c_rad_ has to be considered to account for anticipated significant effects that bulk rheology has on spreading and cooling processes of active lava^24^. Consequently, two TADR estimations were performed with (1) ${c}_{{rad}_{min}}=0.5\times {c}_{rad}$ and (2) ${c}_{{rad}_{max}}=1.5\times {c}_{rad}$. The final TADR is the mean of both.

The erupted lava volume between two sequential satellite acquisitions t_i_ and t_j_ can be calculated by computing the integral between of ${TADR}_{{t}_{i}}$ and ${TADR}_{{t}_{j}}$ (Eq. (3)). The final erupted lava volume V is the cumulative sum of V_t_ (Eq. (4)).

3 $${V}_{t}={\int }_{{t}_{i}}^{{t}_{j}}{TADR}_{t}\left(t\right)dt=0.5\times \left({t}_{j}-{t}_{i}\right)\times \left({TADR}_{{t}_{j}}+{TADR}_{{t}_{i}}\right)$$

4 $$V= \sum {V}_{t}$$

#### Lava volume estimates from multi-sensor satellite data

The lava volume estimates based on the VHR satellite imagery (TanDEM-X and Pléiades) are more reliable than the volume estimates from thermal EO data (cf. detailed explanation in “Results” and “Discussion” sections). Consequently, we used the TanDEM-X volume estimates, that were derived from TanDEM-X acquisitions during the eruption, to calibrate the volume estimates from thermal EO data. This was done by replacing the thermal volume estimates by the value of the next available TanDEM-X volume measurement. The calibrated time series is then the high frequent thermal EO data volume estimates but corrected for more precise measurements (TanDEM-X) whenever available (Fig. 5). The uncertainty of the thermal EO estimates were considered in the final calibrated time series.

## Data Availability

Original satellite data are available via DLR (TanDEM-X) and Airbus/ESA (Pléiades). Information derived from the satellite data are available from the corresponding author on request.
